# Oxygen equilibration dynamics in assisted reproductive technology embryo culture media

**DOI:** 10.1007/s10815-026-03849-7

**Published:** 2026-03-21

**Authors:** Sanjana Kulkarni, Bailey K Morris, Sacha A Krieg, Thomas O’Leary, Adam Krieg

**Affiliations:** 1 Present Address: Division of Reproductive, Endocrinology and Infertility, Department of Obstetrics and Gynecology, Oregon Health and Science Univeristy, OR Portland, United States; 2 Division of Gynecologic Oncology, Department of Obstetrics and Gynecology, Oregon Health and Science University, OR Portland, United States; 3 Division of Reproductive and Developmental Sciences, Oregon National Primate Research Center, OR Beaverton, United States

**Keywords:** ART media preparation, Reactive oxygen species, Oxygen saturation equilibration, Media equilibration

## Abstract

**Purpose:**

Optimal oxygen saturation is essential for successful in vitro embryo culture in assisted reproductive technology (ART). Reduced oxygen levels (3–8%) improve embryo development by minimizing oxidative stress; however, limited knowledge exists about transient oxygen fluctuations during handling outside hypoxic incubators. This study aimed to quantify oxygen saturation dynamics in embryo culture media under conditions designed to replicate routine ART laboratory workflows.

**Methods:**

Embryo culture media droplets were prepared in room air and overlayed with light or heavy mineral oil. Dishes were equilibrated in a hypoxia chamber (5% O_2_, 5% CO_2_, 37 °C), then transferred to an atmospheric incubator (18–19% O_2_, 5% CO_2_) for equilibration, and then this was repeated once more. Oxygen saturation was measured every 30 s using a fiber-optic microprobe. Each experiment was replicated three times, yielding six equilibration and six reoxygenation curves. Data were fit to single-phase exponential models to calculate half-lives and rate constants.

**Results:**

Media was allowed to equilibrate from atmospheric to hypoxic conditions for 12 h. Light oil overlays demonstrated faster equilibration (half-life 71 min) compared to heavy oil (half-life 116 min, *p* < 0.0001). Upon reoxygenation, oxygen saturation rose rapidly, with light oil droplets reoxygenating with a half-time rise of 50 min and heavy oil in 78 min (*p* < 0.0001).

**Conclusion:**

In ART media, hypoxic oxygen equilibration is a gradual process while reoxygenation is rapid. Oil viscosity significantly influences oxygen equilibration dynamics, with light oil permitting faster equilibration and reoxygenation. These findings underscore the importance of minimizing atmospheric exposure during routine handling and highlight the role of overlay oil in reducing transient oxygen fluctuations.

## Introduction

The success of in vitro embryo culture in assisted reproductive technology (ART) is critically dependent on the microenvironmental conditions to which embryos are exposed. A key component of this environment is oxygen, which plays a pivotal role in supporting embryonic development. In vivo, prior to implantation, embryos develop within the oviduct and uterus where the oxygen levels are considerably lower than atmospheric levels of 20–21% O_2_ [1, 2]. In the female reproductive tract, oxygen concentration normally falls within the 2% to 8% O_2_ range [3, 4]. ART laboratories have adopted reduced oxygen culture systems, most commonly around 5% O_2_ to more closely mimic the environment of the female reproductive tract [5, 6]. Multiple comprehensive studies have demonstrated that oxygen tension regulates cellular metabolism and deviations from physiological levels can induce oxidative stress that compromises embryo viability [5, 7].

Excessive oxygen exposure during cell culture has been implicated in impaired embryonic development through oxidative mechanisms [8]. At high oxygen tension (approximately 21% O_2_), reactive oxygen species (ROS) are generated, leading to lipid peroxidation, protein modification, and DNA damage that ultimately compromise embryo viability [9, 10]. Early studies published between 1969 and 1971 demonstrated that culturing mouse embryos in low oxygen improved viability [11, 12]. Studies evaluating other species such as human, bovine, mouse, goat, and pig have consistently shown that culture under hypoxic conditions reduces ROS generation, preserves mitochondrial integrity, and improves blastocyst formation and embryo morphology [13–17]. More recent studies in rhesus macaques have similarly reported that low-oxygen conditions (3–5% O_2_) support follicle growth, survival, and sustained steroidogenic function in vitro compared with atmospheric oxygen [18].

In human embryo culture, the use of low oxygen to improve ART outcomes is generally well accepted. Several randomized controlled trials and meta-analyses have demonstrated improved blastocyst development, implantation, and cumulative live birth rates in human embryos cultured under 5% oxygen compared with atmospheric oxygen [19, 20]. As a result, hypoxic incubation has become more common in many ART laboratories [21]. Despite understanding the importance of hypoxic environments in embryo culture, embryo manipulation often requires exposure to atmospheric conditions. The dynamic nature of oxygen exposure during routine laboratory handling remains poorly understood. While incubators maintain stable hypoxic conditions, embryos are transiently exposed to atmospheric oxygen during key manipulations such as gamete handling, fertilization checks, embryo assessments, and media preparation. These brief exposures may introduce ROS and undermine the protective effects of a low-oxygen environment. A fundamental gap exists in understanding how rapidly oxygen levels change when culture media transition between atmospheric and hypoxic environments. The rate of oxygen equilibrium could impact embryos or oocytes as rapid reoxygenation may lead to prolonged exposure to nonphysiological oxygen concentrations, potentially affecting development and implantation.

The objective of this study is to quantify the time required for ART culture media to equilibrate from atmospheric (21% O_2_) to hypoxic (5% O_2_) conditions and vice versa. There are some prior studies that have established foundational measurements of oxygen dynamics in ART systems, and our work is not the first demonstration of oxygen equilibration in embryo culture media. Our novelty lies in applying high-resolution fiber-optic oxygen sensing to clinically relevant microdroplet cultures and in systematically comparing light versus heavy mineral oil overlays to define viscosity-dependent effects on both hypoxic equilibration and reoxygenation [22, 23]. By characterizing these equilibration times, we aim to provide practical data that may inform laboratory workflows and help minimize potentially harmful oxygen fluctuations during embryo culture.

## Methods/material

### Preparation of culture droplets

Dishes were prepared at atmospheric oxygen levels at approximately 60 m above sea level and at room temperature in a Baker Sterigard class II A/B3 biological safety cabinet under aseptic conditions. Embryo culture medium (GlobalTotal) was dispensed at room temperature into 60-mm polystyrene dishes (Falcon 351,007) in 30-µl sized droplets, 4 microdrops in each dish to mimic standard clinical embryology conditions. After the microdrops were dispensed, they were overlaid with 8 mL of mineral oil with an approximate depth of 5 mm. Dishes were made with two different types of oil, light (Manufacturer: LifeGlobal®, Product Name: LiteOil®, Reference number: LGOL-100 (100 mL); Lot number: LGOL-171121E, medium viscosity for culture and micromanipulation, Regulatory status: CE marked, sterile, Rx only) and heavy (Manufacturer: LifeGlobal®, Product Name: LifeGuard®, Reference number: LGUA-100 (100 mL); Lot number: LGUA-170808E, high viscosity oil, for continuous culture to protect embryos from VOCs, Regulatory status: CE marked, sterile, Rx only) oil. Both oils were stored at room temperature in atmospheric air, and the total storage time prior to use was 1 month. The dishes were made one at a time prior to the addition of the oil to ensure that there was minimal evaporation. Three independent measurements for each series were conducted on separate days. The oil and the media were dispensed at room temperature and were not equilibrated before dish preparation.

### Oxygen measurements

**Fig. 1:**
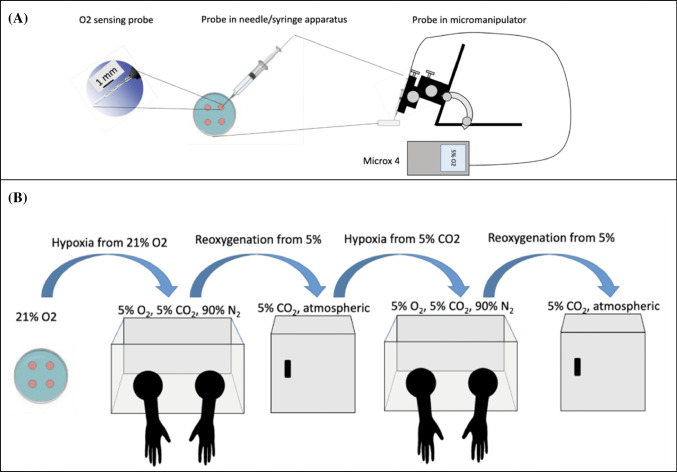
**A** Diagram of oxygen sensing apparatus. Oxygen concentration monitored using Microx 4 oxygen sensor equipped with needle-type probe (Pst-7-flat broken tip, PreSens GmbH, Germany). Probe consists of fiber-optic line capped with oxygen-sensitive sensor foil that changes its reflective angle with O_2_ concentration. Fiber-optic line is threaded through an 18-gauge needle and 1-mL tuberculin syringe. A PreSens manual micromanipulator holds the probe in position with sensor foil immersed in media droplet. Sensor was calibrated to 15% w/v sodium sulfite (0% O_2_) and air saturated water (21% O_2_). **B** Diagram of experimental timelines. After transfer and equilibration of the O_2_ sensor in the hypoxia chamber, plates containing oil-embedded media droplets were transferred to hypoxia glovebox incubators (5% O_2_, 5% CO_2_, ~ 90% N_2_, 37 °C, CoyLabs) for 12–18 h and measured using the Microx 4 as in **A**. After transfer and equilibration of the O_2_ sensor in the standard incubator (5% CO_2_, 37 °C), the dish was transferred to a conventional 5% CO_2_, 37 °C incubator and allowed to reoxygenate overnight (8–12 h). After transfer and equilibration of the O_2_ sensor in the hypoxia chamber, the dish was returned to the hypoxia chamber for 12–18 h. Finally, the O_2_ sensor was transferred and equilibrated in the standard incubator (5% CO_2_, 37 °C); the dish was reoxygenated for 8–12 h. O_2_ measurements were collected every 30 s throughout the duration of the experiments

Changes in oxygen tension were measured every 30 s with a Microx4 oxygen meter equipped with a needle-type fiber-optic oxygen sensing microprobe (PreSens, GmbH). Before any transfer of the culture dishes to the hypoxia chamber or the incubator, the sensor apparatus and micromanipulator were moved to the respective destination chamber/incubator at least 15 min before transferring the dish for measurement, ensuring that the Microx4 thermometer was equilibrated before the oxygen sensor came in contact with the media droplet, and allowing near-instantaneous measurements upon dish transfer. The probe was carefully inserted into the center of a culture droplet under mineral oil, avoiding bubble formation or contact with dish plastic. The sensing probes were mounted in a manual micromanipulator to ensure consistent placement of the sensor in the media droplet (Fig. 1A). The PreSens probes use an oxygen-sensitive foil that does not consume oxygen during measurement.

### Experiment workflow and incubation conditions

Dishes were first created at room temperature in atmospheric oxygen conditions on the benchtop. Dishes were then transferred to a hypoxic glove box incubator (CoyLabs, Inc.) set to 5% O_2_, 5% CO_2_, and 37 °C. The hypoxic glove box was equipped with a continuous oxygen monitoring system to ensure stable gas composition. The chamber’s oxygen sensor was calibrated to pure nitrogen and atmospheric air prior to each measurement series. The droplet oxygen concentration was measured until O_2_ equilibrated after approximately 8–12 h. We chose 12 h for our equilibration period to reflect standard operational practice in human embryo culture laboratories rather than a strictly physiochemical definition of equilibration. Following this, the plate and probe were then removed to a standard cell culture incubator (NuAire TS Auto Flow CO_2_ water-jacketed incubator) equilibrated with atmospheric O_2_ with an actual reading of 18–19% O_2_ and 5% CO_2_ incubator. The single droplets were monitored until saturation was reached (approximately 12 h). Plates and probes were then transferred back to the hypoxic glove box incubator set to 5% O_2_ and 5% CO_2_. The plates were reoxygenated for a final time for approximately 8–12 h (Fig. 1B).

### Replication

The complete experiment was performed three times independently for each oil type, with new dishes, media, and oil preparations. This yielded six hypoxic equilibration curves (three from transitions 21% to 5% O_2_, 5% CO_2_; three from 5% CO_2_ in humidified air). The sequential reoxygenation experiments were treated as independent measurements, resulting in six reoxygenation curves per oil overlay (5% O_2_, 5% CO_2_ to 21% O_2_, 5% CO_2_ in humidified air) (Fig. 1B).

### Data analysis

Oxygen concentration (% O_2_) over time was exported from PreSens software. Curves were fitted to a single-phase exponential decay or single-phase association model in GraphPad Prism software. Data was reported as mean with 95% confidence intervals. Reoxygenation and hypoxic equilibration half-times were calculated using least squares fit algorithm in GraphPad Prism. Equilibration curves were compared using extra sum of squares *F*-test with a *p*-value of < 0.05 considered significant. Reoxygenation dynamics changed quickly for light and heavy oil within the first hour; therefore, further discrete points were reanalyzed at 5-, 10-, and 15-min intervals using one-way ANOVA with corrections for repeated measurements with Dunnett’s multiple comparisons test in GraphPad Prism. Diffusion coefficients for both light oil and heavy oil overlays during reoxygenation and deoxygenation were estimated using the time required to reach 90% of the respective oxygen plateau, using the formulae for single phase decay: $Y=\left(Yo-Yplateau\right)*{e}^{-Kt}+ Yplateau$ and pseudo-first-order association: $Y=Yo+(Yplateau-Yo)(1-{e}^{-Kt})$. *K*, *Y*_o_, and *Y*_plateau_ (*Y* refers to oxygen concentration) constants derived from GraphPad Prism were used to derive the 90% plateau times for reoxygenation and deoxygenation under each oil condition. The diffusion distance (X) was approximated as 5 mm based on pilot measurements of 8 mL of oil in 6-cm plates. Diffusion coefficients (D) were then calculated using the relationship $D={X}^{2}/(2t)$.

## Results

### Hypoxic equilibration of media

**Fig. 2:**
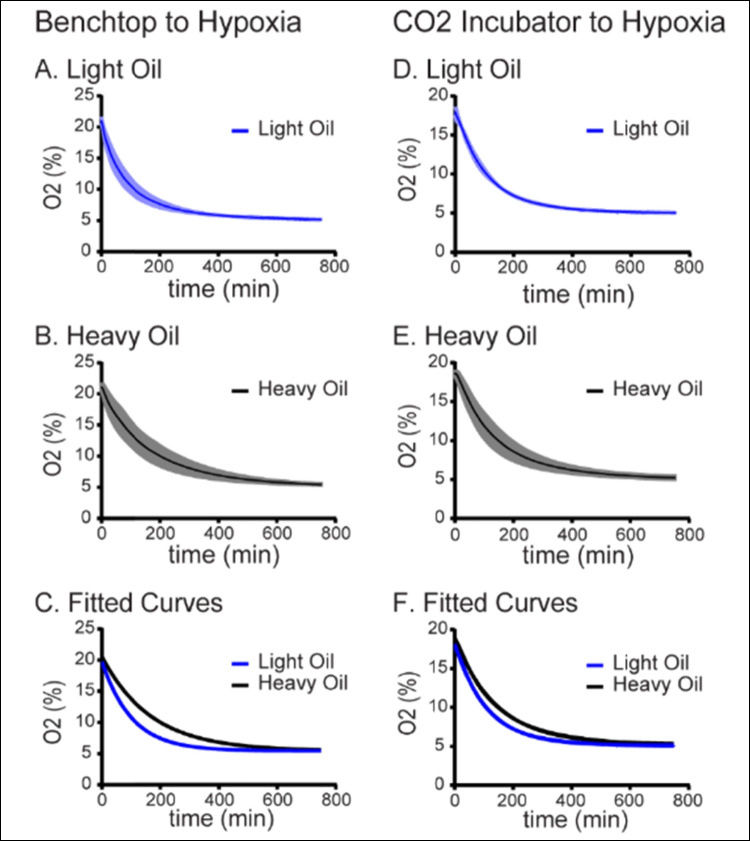
Influence of atmospheric O_2_ and culture oil density on the dynamics of hypoxic equilibrium.** A **Hypoxic equilibration in light oil from atmospheric conditions. Oxygen concentration was measured in embryo culture media overlayed with light oil as described in the “Methods/material” section. The blue line represents the mean oxygen concentration over time after the media was transferred from the benchtop (~ 20% O_2_) into the hypoxia chamber (~ 5% O_2_, *N* = 3 independent replicates, SE shown in light blue). **B **Hypoxic equilibration in heavy oil from atmospheric conditions. Oxygen concentration was measured in embryo culture media overlayed with heavy oil as described in **A**. The black line represents the mean oxygen concentration over time following transfer to the hypoxia chamber (*N* = 3 independent replicates, SE shown in gray). **C **Comparison of atmospheric-to-hypoxia equilibration curves. Data from **A** (blue) and **B** (black) were fit to single-phase decay curves and compared using the extra sum of squares *F*-test as described in the “Methods/material” section (*p* < 0.001). **D **Hypoxic equilibration in light oil from standard incubator conditions. Oxygen concentration was measured in embryo culture media overlayed with light oil. The blue line represents the mean oxygen concentration over time after the media were transferred from a standard incubator (~ 18–19% O_2_) to a hypoxia chamber (*N* = 3 independent replicates, SE shown in light blue). **E **Hypoxic equilibration in heavy oil from standard incubator. The black line represents the mean oxygen concentration over time after media immersed in heavy oil was transferred from a standard incubator to a hypoxia chamber (*N* = 3 independent replicates, SE shown in gray). **F **Comparison of incubator-to-hypoxia equilibration curves. Data from **D** (blue) and **E** (black) were fit to single-phase decay curves and compared using the extra sum of squares *F*-test as described in the “Methods/material” section (*p* < 0.001)

Media droplets immersed in culture oil were equilibrated to hypoxic conditions as described in the “Methods/material” section and illustrated in Fig. 1. When dishes prepared on the benchtop (atmospheric, ~ 21% O2, *n* = 3) were transferred to the hypoxia chamber, complete equilibration to 5% O_2_ took approximately 12 h with light oil overlay (Fig. 2A). Equilibration followed a single-phase decay curve, with a plateau of 5.434% O_2_ and a half-life of 71.03 min (CI 95%; 70.23 to 71.84 min; Table 1). Decay curves for independent replicate experiments using heavy oil immersion (*n* = 3) demonstrated slower trajectory when transferred from atmospheric oxygen to a hypoxia chamber, with a plateau of 5.440% O_2_ and a half-life of 116.3 min (CI 95%; 114.4 to 118.2, Fig. 2B and Table 1). Comparison of the decay curves using the extra sum of squares *F*-test demonstrated a statistically significant difference (*p* < 0.0001) between the light oil (*K* = 0.009759; CI 95% 0.009648 to 0.00987) and heavy oil preparations (*K* = 0.005961; 95% CI 0.005865 to 0.006058; Table 1 and Fig. 2C). Similarly, when equilibrated dishes overlain with light oil were transferred to the hypoxia chamber from a standard humidified 5% CO_2_ incubator set to atmospheric conditions (*n* = 3, ~ 18–19% O_2_), equilibration was also complete in approximately 12 h and followed a single-phase decay curve with a plateau of 5.141% O_2_ and a half-life of 76.76 min (CI 95%; 76.49 to 77.03 min; Fig. 2D and Table 1). When droplets equilibrated in heavy oil were moved from the incubator to the hypoxia chamber (*n* = 3), oxygen measurements also followed a single-phase decay curve with a plateau of 5.311% O_2_ and a half-life of 98.33 min (CI 95%; 97.09 to 99.59, Fig. 2E and Table 1). The equilibration curves for these two series were significantly different (*P* < 0.0001, Fig. 2F and Table 1). The light oil curve had a *K*-value of 0.009030 (CI 95%; 0.0089 to 0.0091) while heavy oil had a *K*-value of 0.007049 (CI 95%; 0.006960 to 0.007139).

**Table 1:** Hypoxia trends

	Light oil	Heavy oil
Atmosphere > 5% O_2_		
Half-life [min]; (95% CI)	71.03; (70.23 to 71.84)	116.3; (114.4 to 118.2)
K-value; (95% CI)	0.0098; (0.0096 to 0.0098)	0.0060; (0.0059 to 0.0061)
Atmospheric incubator > 5% O_2_		
Half-life [min]; (95% CI)	76.76; (76.49 to 77.03)	98.3; (97.09 to 99.59)
K-value; (95% CI)	0.0090; (0.0089 to 0.0091)	0.0071 (0.0070 to 0.0071)

### Reoxygenation dynamics

**Fig. 3:**
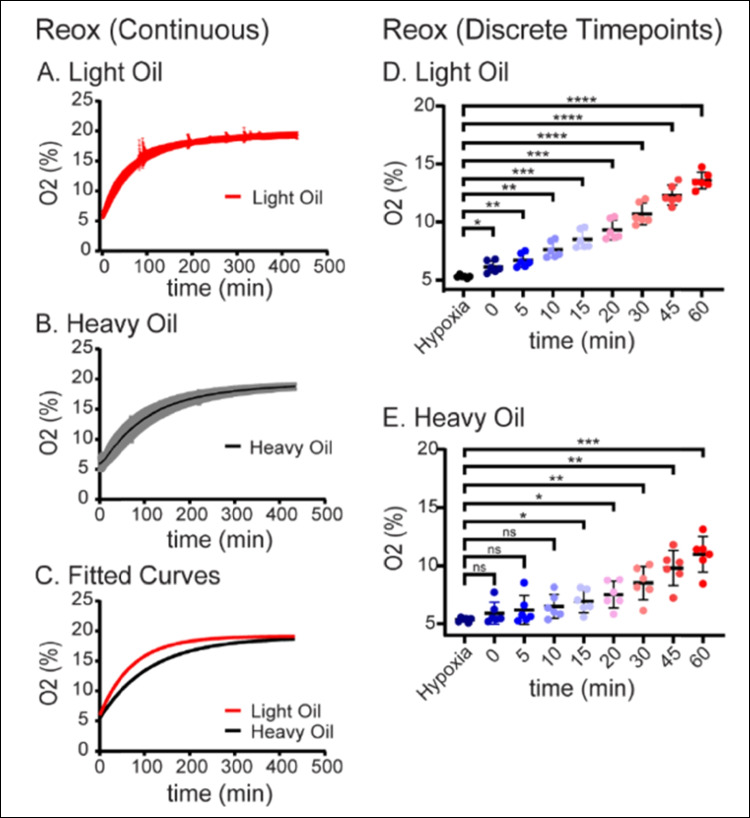
Influence of oil density on dynamics of reoxygenation. **A **Reoxygenation dynamics in light oil. Oxygen concentration was measured in embryo culture media overlayed with light oil as described in the “Methods/material” section. The red line represents the mean oxygen concentration over time after hypoxic media (~ 5% O_2_) transferred into an atmospheric incubator (~ 18–19% O_2_; *N* = 6 independent replicates, SE shown in light red). **B **Reoxygenation dynamics in heavy oil. Oxygen concentration was measured in embryo culture media overlayed with heavy oil. The black line represents the mean oxygen concentration over time after the media was transferred into an atmospheric incubator (~ 18–19% O_2_; *N* = 6 independent replicates, SE shown in gray). **C** Comparison of reoxygenation curves. Data from **A** (light oil, blue) and **B** (heavy oil, black) was fit to single-phase decay curves as described in the “Methods/material” section and compared using extra sum of squares *F*-test as described in the “Methods/material” section (*p* < 0.001). **D **Reoxygenation at discrete time points in light oil. Average O_2_% measurements were plotted at discrete timepoints for up to 60 min following reoxygenation of embryo culture overlain in light oil. Data represents the mean concentration ± 95% confidence intervals compared to hypoxia using one-way ANOVA of repeated measurements with Dunnet’s multiple comparison test (* = *p* < 0.05, ** = *p* < 0.01, *** = *p* < 0.001, **** = *p* < 0.0001). **E **Reoxygenation at discrete points in heavy oil. Data represents the mean concentration ± 95% confidence intervals compared to hypoxia using one-way ANOVA of repeated measurements with Dunnet’s multiple comparison test (* = *p* < 0.05, ** = *p* < 0.01, *** = *p* < 0.001)

In both light oil and heavy oil media preparations, media were allowed to reach complete reoxygenation for approximately 12 h. The half-time rise for reoxygenation in the light oil was 50.22 min (CI 95%; 49.63 to 50.82 min) (Table 2). Reoxygenation dynamics of light oil (*n* = 6) demonstrated a single-phase association curve with *K*-value of 0.01380 (CI 95%; 0.01364 to 0.01296; Fig. 3A and Table 2). Comparatively, the reoxygenation half-time rise for heavy oil was 78.49 min (CI 95%; 77.07 to 79.95; Table 2). The reoxygenation of the droplets immersed in heavy oil also demonstrated a single-phase association curve with a *K*-value of 0.008831 (CI 95%; 0.008670 to 0.008993; Table 2 and Fig. 3B). Comparison of reoxygenation curves with the extra sum of squares *F*-test demonstrated significantly slower reoxygenation dynamics for the heavy oil dishes (*p* < 0.0001; Fig. 3C and Table 2).

**Table 2:** Reoxygenation trends

	Light oil	Heavy oil
5% O_2_ > atmospheric incubator		
Half-time rise [min]; (95% CI)	50.22; (49.63 to 50.82)	78.49; (77.07 to 79.95)
K-value; (95% CI)	0.0138; (0.0136 to 0.0129)	0.0088; (0.0087 to 0.0090)

Reoxygenation of media overlain by light oil in the humified incubator (~ 18–19% O_2_) was statistically significant immediately following transfer (t = 0 min), with a mean difference of − 0.8330 compared to hypoxic equilibration (CI 95%; − 1.487 to − 0.1794) and a *p*-value of 0.0191 (Fig. 3D and Table 3). The reoxygenation of media under heavy oil was at a slower rate than the light oil with statistically significant reoxygenation occurring 15 min after transfer from hypoxia, with a mean difference of − 1.590 (CI 95%; − 2.944 to − 0.2362) and a *p*-value of 0.026 (Fig. 3D and Table 4).

**Table 3:** Light oil reoxygenation discrete points

Dunnett’s multiple comparisons test	Mean diff	95.00% CI of diff	Below threshold	Adjusted *p*-value
Hypoxia vs. 0 (%)	− 0.833	− 1.487 to − 0.179	Yes	0.0191
Hypoxia vs. 5 (%)	− 1.385	− 2.169 to − 0.600	Yes	0.0046
Hypoxia vs. 10 (%)	− 2.301	− 3.279 to − 1.324	Yes	0.0012
Hypoxia vs. 15 (%)	− 3.197	− 4.335 to − 2.059	Yes	0.0005
Hypoxia vs. 20 (%)	− 4.009	− 5.258 to − 2.761	Yes	0.0003
Hypoxia vs. 30 (%)	− 5.392	− 6.751 to − 4.033	Yes	< 0.0001
Hypoxia vs. 45 (%)	− 7.004	− 8.245 to − 5.764	Yes	< 0.0001
Hypoxia vs. 60 (%)	− 8.263	− 9.296 to − 7.229	Yes	< 0.0001

**Table 4:** Heavy oil reoxygenation discrete points

Dunnett’s multiple comparisons test	Mean diff	95.00% CI of diff	Below threshold	Adjusted *P*-value
Hypoxia vs. 0 (%)	− 0.563	− 1.973 to 0.846	No	0.5646
Hypoxia vs. 5 (%)	− 0.843	− 2.678 to 0.994	No	0.4514
Hypoxia vs. 10 (%)	− 1.149	− 2.604 to 0.306	No	0.1140
Hypoxia vs. 15 (%)	− 1.590	− 2.944 to − 0.236	Yes	0.0268
Hypoxia vs. 20 (%)	− 2.163	− 3.770 to − 0.555	Yes	0.0153
Hypoxia vs. 30 (%)	− 3.157	− 5.215 to − 1.099	Yes	0.0087
Hypoxia vs. 45 (%)	− 4.459	− 6.662 to − 2.256	Yes	0.0025
Hypoxia vs. 60 (%)	− 5.638	− 7.860 to − 3.417	Yes	0.0009

### Diffusion coefficient and time to 90% plateau

During reoxygenation, light oil demonstrated a faster equilibration, reaching 90% of the oxygen plateau (T_90%_) at 140.04 min with a higher diffusion coefficient (1.29 × 10^−^⁹) compared with heavy oil, which exhibited a prolonged T_90%_ of 222.51 min and a lower diffusion coefficient (9.36 × 10^−1^⁰) (Table 5). Similar trends were observed during deoxygenation, both under atmospheric exposure and within the incubator. Under atmospheric deoxygenation, light oil reached T_90%_ at 335.42 min with a diffusion coefficient of 6.21 × 10^−1^⁰, whereas heavy oil showed markedly slower kinetics (T_90%_558.10 min; diffusion coefficient 3.73 × 10^−1^⁰). Within the incubator, deoxygenation remained slower with heavy oil (T_90%_ 460.66 min; diffusion coefficient 4.52 × 10^−1^⁰) compared with light oil (T_90%_ 357.89 min; diffusion coefficient 5.82 × 10^−1^⁰). By contrast, when we used the published diffusion coefficient for oxygen through 0.5 cm of paraffin oil (1.67 × 10^−9^ m^2^/s, calculated by Stokes 2009), the approximate time to diffusion was estimated to be 125 min, which was faster than the D_t90%_ for light oil, reflecting its lower viscosity [22].

**Table 5:** Diffusion coefficient and time to 90% plateau

	Reoxygenation		Deoxygenation (atmosphere)		Deoxygenation (incubator)	
	T_90%_ (minutes)	Diffusion coefficient (t_90_) (m^2^/sec)	T_90%_ (minutes)	Diffusion coefficient (t_90_) (m^2^/sec)	T_90%_ (minutes)	Diffusion coefficient (t_90_) (m^2^/sec)
Light oil	140.04	1.29E-09	335.42	6.21E-10	357.89	5.82E-10
Heavy oil	222.51	9.36E-10	558.10	3.73E-10	460.66	4.52E-10

## Discussion

To the best of our knowledge, our study expands on the direct quantitation of oxygen equilibration and reoxygenation dynamics in embryo culture media using conditions designed to mimic routine ART laboratory practices. We demonstrated that oxygen exchange is gradual but predictable with equilibration under hypoxia. Our results also demonstrate that reoxygenation can occur rapidly with significant rises in O_2_ within minutes of exposure to ambient air. Additionally, the choice of oil overlay significantly alters these dynamics, with light oil allowing faster equilibration and reoxygenation compared to heavy oil. The influence of oil type impacts buffering capacity and reoxygenation rates, creating a dilemma in oil choice for embryo culture.

The intent of our experimental design was to closely reflect practices in embryology laboratories. During routine in vitro fertilization (IVF) procedures, media preparation is frequently performed on the benchtop in atmospheric oxygen, after which plates are transferred into low-oxygen incubators to more closely mimic the physiologic environment in utero. The first portion of our experiment closely emulated this, as the dishes were prepared on the benchtop then moved to a hypoxic incubator (Fig. 1B). Similarly, oocytes and embryos are often removed from the hypoxic incubator for micromanipulation steps such as intracytoplasmic sperm injection (ICSI), embryo scoring, or embryo biopsy, briefly exposing them to atmospheric oxygen before being returned to reduced oxygen [24]. Since embryo manipulation often takes place in atmospheric oxygen, to recreate the laboratory environment, the O_2_ sensor was equilibrated in the destination incubator; dishes were then transferred to a conventional 5% CO_2_, 37 °C incubator, and allowed to equilibrate overnight to atmospheric conditions to measure reoxygenation dynamics of embryo culture media (Fig. 1B).

In standard IVF practice, oocytes and embryos are frequently removed from hypoxic incubators for micromanipulation steps such as ICSI, embryo scoring, or biopsy, briefly exposing them to atmospheric oxygen before returning them to reduced oxygen conditions. These procedures are intentionally brief, typically lasting between 5 and 15 min, to minimize environmental fluctuations. Studies have emphasized that even short exposures outside the incubator can impact temperature and gas stability [25, 26]. By aligning our oxygen measurements with these common workflow intervals, we underscore the practical relevance of our findings and highlight the importance of minimizing exposure time during routine culture operations. This strengthens the practical relevance of our findings and underscores the importance of minimizing exposure time during routine culture operations.

Some ART labs perform their dish preparation in a preequilibrated incubator (18–19% O_2_) and then move them into a hypoxic incubator. Therefore, we assessed hypoxia equilibrium when embryo culture media was moved from an atmospheric incubator to a hypoxia chamber (5% O_2_). In our experiment, the dishes were then returned to the hypoxia chamber for at least 12 h and then reoxygenated to mimic the routine handling steps, allowing us to quantify the equilibration dynamics of culture media under realistic laboratory conditions.

In our study, we used a hypoxic glove box incubator (hypoxia chamber) maintained at 5% O_2_ which prevented reoxygenation artifacts during the process of manipulating the oxygen probe. This chamber also maintained constant temperature, which can also influence oxygen sensor readings [4]. The primary goal of our study was to mimic culture conditions without inserting the additional influence of embryo metabolism. Our work highlights how fluctuations in environmental oxygen saturation could influence the stability of the microenvironment surrounding embryos. By characterizing the kinetics of oxygen equilibration across different manipulations, our study underscores the potential for transient reoxygenation events to affect embryo culture outcomes. This provides valuable insight into best practices for minimizing oxygen fluctuations, reinforcing the importance of consistent hypoxic culture conditions in optimizing IVF success rates.

Both the light and heavy oil equilibrations followed single-phase decay curves, but the half-life to equilibration in light oil was significantly lower than the half-life for the heavy oil. These results indicate that longer equilibration times should be considered in standard laboratory workflows that use heavy oil. This highlights the impact of oil viscosity and density on oxygen diffusion, which had been suggested in theory but here is directly measured [27]. Our study further highlights the critical role of oil overlay viscosity in shaping oxygen diffusion dynamics within embryo culture systems. Across all experimental conditions, heavy oil consistently slowed oxygen equilibration and reduced effective diffusion coefficients compared with light oil, indicating a greater diffusion barrier at the oil-media interface. Notably, this effect was evident during both reoxygenation and deoxygenation, suggesting that heavy oil not only delays recovery from atmospheric exposure but also slows changes in oxygen tension once embryo culture dishes are returned to controlled environments. The gradual pace of change may be perceived as protective; the markedly prolonged T_90%_ observed with heavy oil, particularly under atmospheric deoxygenation, raises concern that embryos may experience extended periods of nonphysiologic oxygen tension following routine handling. In contrast, light oil permitted more rapid equilibration, more closely approximating intended incubator oxygen conditions. Together, these data suggest that oil selection is a nontrivial determinant of the embryonic oxygen microenvironment and may have important implications for optimizing culture conditions and minimizing unintended oxidative stress during assisted reproductive procedures.

Our data analysis also demonstrated a variability in diffusion coefficients across reoxygenation and deoxygenation conditions indicating that oxygen transfer in embryo culture is not solely governed by a single intrinsic constant but rather reflects context-dependent kinetics. Both light and heavy oil demonstrated direction-dependent differences, with faster diffusion during reoxygenation compared to deoxygenation, likely driven by steeper oxygen gradients during atmospheric exposure (5% to 21% O_2_) than during return to hypoxia (21% to 5% O_2_). The comparatively slower deoxygenation may also suggest that the culture media itself exhibits oxygen retention properties, potentially related to dissolved gas solubility and limited mixing within the microdroplet environment, which may help transiently buffer oxygen fluctuations and maintain conditions supportive of embryo metabolic demands. Heavy oil consistently exhibited longer T_90%_ values and lower effective diffusion coefficients across all conditions, confirming greater resistance to oxygen flux. Importantly, differences between deoxygenation from atmosphere versus incubator further suggest that gas composition and boundary conditions influence equilibration dynamics. Together, these findings support a model in which oxygen tension within embryo culture droplets is an emergent property of oil permeability, gradient magnitude, and environmental context, rather than a fixed parameter dictated solely by incubator settings.

The comparatively slower deoxygenation kinetics may also suggest that the culture media itself exhibits oxygen retention properties, potentially related to dissolved gas solubility or limited convective mixing within the microdroplet environment. However, the proprietary nature of most embryo culture media makes dissection of this phenomenon difficult. Heavy oil consistently exhibited longer T90% values and lower effective diffusion coefficients across all conditions, confirming greater resistance to oxygen flux and enhanced buffering capacity. Differences between deoxygenation from atmosphere versus incubator further indicate that gas composition and boundary conditions influence equilibration dynamics. Together, these findings support a model in which oxygen tension within embryo culture droplets is an emergent property of oil permeability, media characteristics, gradient magnitude, and environmental context, rather than a fixed parameter dictated solely by incubator settings.

The result of our study has relevant implications for ART practice. Notably, oxygen concentrations used for embryo culture vary considerably across clinics and regions. Many laboratories continue to culture embryos at atmospheric oxygen (20%) despite recommendations supporting reduced oxygen conditions (5%) to more closely reflect the physiologic reproductive environment [28, 29]. Culturing embryos at elevated oxygen has been shown to cause massive gene deregulation in mice, including genes required for cell growth and gastrulation [30]. Similarly, bovine embryos cultured under elevated oxygen exhibit increased DNA methylation and reduced blastocyst formation rates [30, 31]. Exposure to atmospheric oxygen further enhances the activity of oxygen-dependent enzymes, leading to accelerated ROS generation, heightened oxidative stress, and prolonged embryo development [7, 32]. This dynamic is particularly concerning given the known sensitivity of preimplantation embryos to oxygen fluctuations and the potential for reactive oxygen species (ROS) mediated damage [33]. ROS are often generated in vitro and rise if oxygen tensions of the media increase, impairing embryo competence and viability [34, 35]. 

Our study has some limitations. First, our kinetic data was derived from a media-only system without the influence of embryo metabolism. Embryos may alter oxygen dynamics due to metabolic waste production and cellular oxygen consumption, which may accelerate or delay equilibration rates [33]. Investigating oxygen kinetics in the presence of embryos**,** as well as directly measuring ROS levels**,** could provide more biologically relevant insights. Furthermore, only one formulation of culture media was tested; therefore, this data may not be general for all other media types. Further studies would be recommended to compare multiple media types. Additionally, media droplets were pipetted into the dish to mimic standard embryo culture conditions, but only one of the droplets could be measured at one time due to the oxygen monitor only having one sensor input. Attempts to measure changes in all four droplets in sequence resulted in equilibration artifacts due to the transition of the sensor between droplets. More consistent readings were achieved by fixing the probe in one droplet with a micromanipulator (Fig. 1A). Incubator space also did not allow for more than one micromanipulator per experiment. Finally, our study did not evaluate the impact of the shift in oxygenation on the embryo itself. It is not yet known if short-term shifts in oxygen have an impact on embryo quality.

The novelty of our studies lies in the fact that it builds on and extends prior work that looks at the oxygen dynamics in ART systems by focusing on clinically relevant microdroplet culture systems. Furthermore, our study directly measures oxygen dynamics with high-resolution fiber-optic microprobe technology. Some prior studies have provided important foundational insights into oxygen dynamics in ART systems but have addressed only select components of the culture microenvironment. Previously, oxygen equilibration dynamics of paraffin oil with mouse embryos in a culture capillary were studied; however, these experiments did not capture real-time oxygen dynamics within microdroplet culture systems under conditions that mirror routine ART workflows [22]. Another study demonstrated that oxygen equilibration in culture media is a slow, hours-long process; however, their work was performed by a handheld blood gas analyzer without an oil overlay and did not evaluate the effects of repeated transitions between hypoxic and atmospheric environments or the role of oil properties in modulating oxygen kinetics [23].

Rather than attempting to establish the first demonstration of oxygen equilibration in ART media, we frame our contribution around real-time quantification of oxygen equilibration and reoxygenation in microdroplet culture conditions that closely mimic routine laboratory handling, direct comparison of light versus heavy mineral oil to define viscosity-dependent effects on oxygen diffusion kinetics, and characterization of the bidirectional and asymmetric nature of hypoxic equilibration versus reoxygenation. By integrating precise oxygen sensing with microdroplet culture and oil overlay comparisons, our work provides a more operationally and physiologically relevant understanding of how laboratory practices and material choices shape the true oxygen microenvironment experienced by embryos during ART.

Taken together, these results argue strongly for minimizing atmospheric exposure during all phases of embryo culture and for implementing hypoxic workstations or isolette wherever feasible. Moreover, our direct measurements of equilibration kinetics offer embryologists practical guidance on how quickly reoxygenation occurs and how oil overlay choices modulate this process. By defining these parameters, our study provides practical and quantitative benchmarks for reoxygenation kinetics that contribute to the development of evidence-based best practices aimed at reducing oxygen-related artifacts, safeguarding embryo development, and ultimately improving IVF outcomes.

## Data Availability

All data supporting the findings of this study are available within the paper.

## References

[1] Fischer B, Bavister BD. Oxygen tension in the oviduct and uterus of rhesus monkeys, hamsters and rabbits. J Reprod Fertil. 1993;99(2):673–9. 8107053 10.1530/jrf.0.0990673

[2] Maas DH, Storey BT, Mastroianni L Jr. Oxygen tension in the oviduct of the rhesus monkey (*Macaca mulatta*). Fertil Steril. 1976;27(11):1312–7. 10.1016/s0015-0282(16)42201-6 824161

[3] Sciorio R, Smith GD. Embryo culture at a reduced oxygen concentration of 5%: a mini review. Zygote. 2019;27(6):355–61. 31544720 10.1017/S0967199419000522

[4] Ng KYB, et al. In vivo oxygen, temperature and pH dynamics in the female reproductive tract and their importance in human conception: a systematic review. Hum Reprod Update. 2018;24(1):15–34. 29077897 10.1093/humupd/dmx028

[5] Mantikou E, et al. Low oxygen concentrations for embryo culture in assisted reproductive technologies. Hum Reprod Update. 2013;19(3):209. 10.1093/humupd/dms055 23377864

[6] Bontekoe S, et al. Low oxygen concentrations for embryo culture in assisted reproductive technologies. Cochrane Database Syst Rev. 2012;2012(7):CD008950. 10.1002/14651858.CD008950.pub2 PMC11683526 22786519

[7] Guerin P, El Mouatassim S, Menezo Y. Oxidative stress and protection against reactive oxygen species in the pre-implantation embryo and its surroundings. Hum Reprod Update. 2001;7(2):175–89. 11284661 10.1093/humupd/7.2.175

[8] Bedaiwy MA, et al. Differential growth of human embryos in vitro: role of reactive oxygen species. Fertil Steril. 2004;82(3):593–600. 15374701 10.1016/j.fertnstert.2004.02.121

[9] Catt JW, Henman M. Toxic effects of oxygen on human embryo development. Hum Reprod. 2000;15(Suppl 2):199–206. 11041525 10.1093/humrep/15.suppl_2.199

[10] Agarwal A, et al. Oxidative stress and assisted reproduction: a comprehensive review of its pathophysiological role and strategies for optimizing embryo culture environment. Antioxidants (Basel). 2022. 10.3390/antiox11030477. 35326126 10.3390/antiox11030477 PMC8944628

[11] Wiesener MS, et al. Widespread hypoxia-inducible expression of HIF-2alpha in distinct cell populations of different organs. FASEB J. 2003;17(2):271–3. 12490539 10.1096/fj.02-0445fje

[12] Whitten WK. The effect of oxygen on cleavage of mouse eggs. In2nd Annual Meeting, Society for the Study of Reproduction, Davis, California 1969 (Vol. 29).

[13] Houghton FD. Hypoxia and reproductive health: hypoxic regulation of preimplantation embryos: lessons from human embryonic stem cells. Reproduction. 2021;161(1):F41-51. 33258799 10.1530/REP-20-0322

[14] Fujitani Y, et al. Effect of oxygen concentration and free radicals on in vitro development of in vitro-produced bovine embryos. J Anim Sci. 1997;75(2):483–9. 9051472 10.2527/1997.752483x

[15] Meuter A, et al. Markers of cellular senescence are elevated in murine blastocysts cultured in vitro: molecular consequences of culture in atmospheric oxygen. J Assist Reprod Genet. 2014;31(10):1259–67. 25106938 10.1007/s10815-014-0299-8 PMC4171413

[16] Batt PA, Gardner DK, Cameron AW. Oxygen concentration and protein source affect the development of preimplantation goat embryos in vitro. Reprod Fertil Dev. 1991;3(5):601–7. 1788401 10.1071/rd9910601

[17] Karja NW, et al. Effects of oxygen tension on the development and quality of porcine in vitro fertilized embryos. Theriogenology. 2004;62(9):1585–95. 15511546 10.1016/j.theriogenology.2004.03.012

[18] Wang K, et al. Exploring the dynamics of follicle development and hormone synthesis: the role of oxygen tension in rhesus macaque follicle culture. bioRxiv. 2025. 10.1101/2025.05.06.652505.41982337

[19] Chen L, et al. Oxygen concentration from days 1 to 3 after insemination affects the embryo culture quality, cumulative live birth rate, and perinatal outcomes. J Assist Reprod Genet. 2023;40(11):2609–18. 37728792 10.1007/s10815-023-02943-4 PMC10643741

[20] Waldenstrom U, et al. Low-oxygen compared with high-oxygen atmosphere in blastocyst culture, a prospective randomized study. Fertil Steril. 2009;91(6):2461–5. 18554591 10.1016/j.fertnstert.2008.03.051

[21] ESHRE Guideline Group on Good Practice in IVF Labs, De los Santos MJ, Apter S, Coticchio G, Debrock S, Lundin K, Plancha CE, Prados F, Rienzi L, Verheyen G, Woodward B. Revised guidelines for good practice in IVF laboratories (2015). Human Reproduction. 2016 Apr 1;31(4):685-6. 10.1093/humrep/dew016 26908842

[22] Stokes YM. Quantifying oxygen diffusion in paraffin oil used in oocyte and embryo culture. Mol Reprod Dev. 2009;76(12):1178–87. 10.1002/mrd.21089 19672876

[23] Glage ED, Casey; Gunasegaran K, Sorby K, The race to equilibrate. Fertility and Reproduction, 2022. **4**: p. 212.

[24] Wale PL, Gardner DK. The effects of chemical and physical factors on mammalian embryo culture and their importance for the practice of assisted human reproduction. Hum Reprod Update. 2016;22(1):2–22. 26207016 10.1093/humupd/dmv034

[25] Morbeck DE, Baumann NA, Oglesbee D. Composition of single-step media used for human embryo culture. Fertil Steril. 2017;107(4):1055-1060 e1. 28238490 10.1016/j.fertnstert.2017.01.007

[26] Swain JE. Decisions for the IVF laboratory: comparative analysis of embryo culture incubators. Reprod Biomed Online. 2014;28(5):535–47. 24656561 10.1016/j.rbmo.2014.01.004

[27] Martinez CA, et al. The overlaying oil type influences in vitro embryo production: differences in composition and compound transfer into incubation medium between oils. Sci Rep. 2017;7(1):10505. 10.1038/s41598-017-10989-5. 28874873 10.1038/s41598-017-10989-5 PMC5585310

[28] Christianson MS, et al. Embryo catheter loading and embryo culture techniques: results of a worldwide web-based survey. J Assist Reprod Genet. 2014;31(8):1029–36. 24913025 10.1007/s10815-014-0250-z PMC4130946

[29] Du Plessis SS, Makker K, Desai NR, Agarwal A. Impact of oxidative stress on IVF. Expert review of obstetrics & gynecology. 2008Jul 1;3(4):539–54.

[30] Belli M, et al. The effect of low and ultra-low oxygen tensions on mammalian embryo culture and development in experimental and clinical IVF. Syst Biol Reprod Med. 2020;66(4):229–35. 32379506 10.1080/19396368.2020.1754961

[31] Li W, et al. High oxygen tension increases global methylation in bovine 4-cell embryos and blastocysts but does not affect general retrotransposon expression. Reprod Fertil Dev. 2016;28(7):948–59. 25515369 10.1071/RD14133

[32] Kovacic B, Vlaisavljevic V. Influence of atmospheric versus reduced oxygen concentration on development of human blastocysts in vitro: a prospective study on sibling oocytes. Reprod Biomed Online. 2008;17(2):229–36. 18681997 10.1016/s1472-6483(10)60199-x

[33] Cooke MS, et al. Oxidative DNA damage: mechanisms, mutation, and disease. FASEB J. 2003;17(10):1195–214. 12832285 10.1096/fj.02-0752rev

[34] Cobley JN. Mechanisms of mitochondrial ROS production in assisted reproduction: the known, the unknown, and the intriguing. Antioxidants. 2020S;9(10):933. 33003362 10.3390/antiox9100933 PMC7599503

[35] Shields HJ, Traa A, Van Raamsdonk JM. Beneficial and detrimental effects of reactive oxygen species on lifespan: a comprehensive review of comparative and experimental studies. Front Cell Dev Biol. 2021;9:628157. 33644065 10.3389/fcell.2021.628157 PMC7905231

